# 1,3-Bis{(+)-(*S*)-[1-(1-naphth­yl)eth­yl]imino­meth­yl}benzene dichloro­methane solvate

**DOI:** 10.1107/S1600536809017528

**Published:** 2009-05-20

**Authors:** Armando Espinosa Leija, Guadalupe Hernández, Sandra Cruz, Sylvain Bernès, René Gutiérrez

**Affiliations:** a Facultad de Ciencias Químicas, UANL, Licenciatura en Química Industrial, Ciudad Universitaria, Monterrey, NL, Mexico; b Laboratorio de Síntesis de Complejos, Facultad de Ciencias Químicas, BUAP, AP 1067, 72001 Puebla, Pue., Mexico; c DEP Facultad de Ciencias Químicas, UANL, Guerrero y Progreso S/N, Col. Treviño, 64570 Monterrey, NL, Mexico

## Abstract

In the title compound, C_32_H_28_N_2_·CH_2_Cl_2_,  the complete Schiff base and solvent molecules are both  generated by crystallographic twofold axes, with the two C atoms of the former and the C atom of the latter lying on the rotation axis. The central benzene ring is substituted with two chiral groups including imine functionalities, with the common *E* configuration. The dihedral angle between the central benzene ring and the terminal naphthalene ring is 45.42 (9)° and that between the two naphthalene rings is 89.16 (8)°. The conformation of the Schiff base allows solvent mol­ecules to fill the voids in the crystal, affording a stable 1:1 solvate, but the solvent inter­acts poorly with the Schiff base, as reflected by its rather high displacement parameters.

## Related literature

For solvent-free synthesis in organic chemistry, see: Jeon *et al.* (2005 ▶); Noyori (2005 ▶); Tanaka & Toda (2000 ▶). For related chiral Schiff bases synthesized using similar routes, see: Tovar *et al.* (2007 ▶).
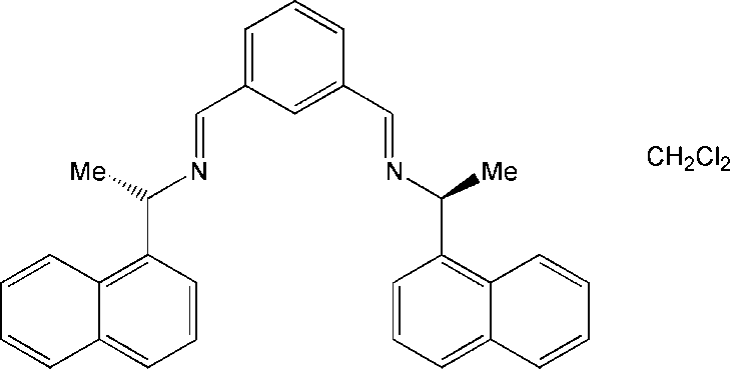


## Experimental

### 

#### Crystal data

C_32_H_28_N_2_·CH_2_Cl_2_
*M* *_r_* = 525.49
Orthorhombic, 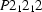

*a* = 8.550 (2) Å
*b* = 20.706 (6) Å
*c* = 7.972 (3) Å
*V* = 1411.3 (7) Å^3^
*Z* = 2
Mo *K*α radiation
μ = 0.25 mm^−1^
*T* = 298 K
0.40 × 0.24 × 0.20 mm

#### Data collection

Siemens P4 diffractometer
Absorption correction: gaussian (*XSCANS*; Siemens, 1996 ▶) *T*_min_ = 0.933, *T*_max_ = 0.954
6308 measured reflections
2497 independent reflections
1428 reflections with *I* > 2σ(*I*)
*R*_int_ = 0.060
3 standard reflections every 97 reflections intensity decay: 2.3%

#### Refinement

*R*[*F* ^2^ > 2σ(*F* ^2^)] = 0.060
*wR*(*F* ^2^) = 0.186
*S* = 1.06
2497 reflections
172 parameters
H-atom parameters constrained
Δρ_max_ = 0.19 e Å^−3^
Δρ_min_ = −0.16 e Å^−3^
Absolute structure: Flack (1983 ▶), 1028 Friedel pairs
Flack parameter: 0.0 (2)

### 

Data collection: *XSCANS* (Siemens, 1996 ▶); cell refinement: *XSCANS*; data reduction: *XSCANS*; program(s) used to solve structure: *SHELXS97* (Sheldrick, 2008 ▶); program(s) used to refine structure: *SHELXL97* (Sheldrick, 2008 ▶); molecular graphics: *Mercury* (Macrae *et al.*, 2006 ▶); software used to prepare material for publication: *SHELXL97*.

## Supplementary Material

Crystal structure: contains datablocks I, global. DOI: 10.1107/S1600536809017528/fj2198sup1.cif

Structure factors: contains datablocks I. DOI: 10.1107/S1600536809017528/fj2198Isup2.hkl

Additional supplementary materials:  crystallographic information; 3D view; checkCIF report

## Figures and Tables

**Fig. 1.**
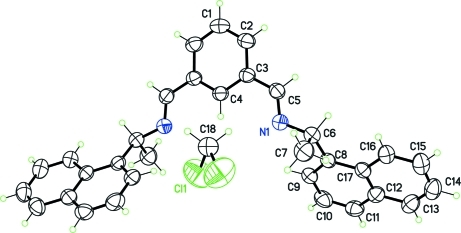
Molecular structure of the title compound, with 30% probability level displacement ellipsoids for non-H atoms. Non-labeled atoms are generated through the symmetry operation 2 - x, 1 - y, z.

**Fig. 2.**
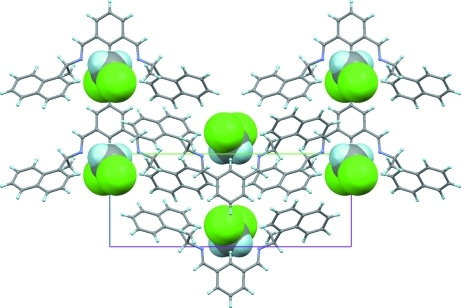
A part of the crystal structure of the title compound along [100], showing the lattice solvent molecules with a space-filling representation.

## supplementary crystallographic information

### Comment

During the last few decades, a central objective in synthetic organic chemistry has been to develop greener and more economically competitive processes for the efficient synthesis of compounds with potential applications in diverse fields. In this context, the solvent-free approach is simple with amazing versatility because it reduces the use of organic solvents and minimizes the formation of other waste. Likewise, the reactions occur under mild conditions and usually require easier workup procedures and simpler equipment. Moreover, it may allow access to compounds that require harsh reaction conditions under traditional approaches or when the yields are too low to be of practical convenience (Jeon *et al.*, 2005; Noyori, 2005; Tanaka & Toda, 2000).

On the other hand, bisimines have lately attracted much attention, mostly due to their versatile coordination behavior and the interesting properties of their metal complexes. These compounds are particularly interesting since they can potentially act in a variety of coordination modes.

Continuing our work on the synthesis of chiral imines (Tovar *et al.*, 2007), we synthesized the title Schiff base under solvent-free conditions and report here its X-ray structure. The asymmetric unit contains one half-molecule and one half dichloromethane molecule, both placed on binary axis (Fig. 1). This arrangement is probably favored by the presence of a chiral center, C6, allowing to orient the substituents of the imine functionality towards the opposite faces of the central benzene core. The crystal is further stabilized by the inclusion of lattice solvent, resulting in a 1:1 solvate. Indeed, the shape of the Schiff base is suitable for the formation of a guest-host complex (Fig. 2). However, as no efficient hydrogen bonds are formed, the solvent molecule presents high displacement parameters, compared to the host (See Fig. 1).

### Experimental

Under solvent-free conditions, a mixture of benzene-1,3-dicarboxaldehyde (0.12 g, 0.9 mmol) and (*S*)-(-)-1-naphthylethylamine (0.32 g, 1.8 mmol) were mixed at 298 K, giving a white solid. The crude material was recrystallized from CH_2_Cl_2_, affording colorless crystals of the title solvate (Yield: 98%; m.p. 343–345 K. [α]^25^_D_=+253.7 (*c*=1, CHCl_3_). IR and NMR data are consistent with the X-ray structure (see archived CIF).

### Refinement

All H atoms were placed in idealized positions and refined as riding to their carrier C atoms, with bond lengths fixed to 0.93 (aromatic CH), 0.96 (methyl CH_3_), 0.97 (methylene CH_2_) and 0.98 Å (methine CH). isotropic displacement parameters were calculated as *U*_iso_(H) = 1.5*U*_eq_(carrier atom) for the methyl group and *U*_iso_(H) = 1.2*U*_eq_(carrier atom) otherwise.

### Crystal data

**Table:**

C_32_H_28_N_2_·CH_2_Cl_2_	*D*_x_ = 1.237 Mg m^−^^3^
*M**_r_* = 525.49	Melting point: 343 K
Orthorhombic, *P*2_1_2_1_2	Mo *K*α radiation, λ = 0.71073 Å
Hall symbol: P 2 2ab	Cell parameters from 51 reflections
*a* = 8.550 (2) Å	θ = 4.0–11.9°
*b* = 20.706 (6) Å	µ = 0.25 mm^−^^1^
*c* = 7.972 (3) Å	*T* = 298 K
*V* = 1411.3 (7) Å^3^	Prism, yellow
*Z* = 2	0.40 × 0.24 × 0.20 mm
*F*(000) = 552	

### Data collection

**Table:**

Siemens P4 diffractometer	1428 reflections with *I* > 2σ(*I*)
Radiation source: fine-focus sealed tube	*R*_int_ = 0.060
graphite	θ_max_ = 25.0°, θ_min_ = 2.0°
2θ/ω scans	*h* = −10→10
Absorption correction: gaussian (*XSCANS*; Siemens, 1996)	*k* = −24→24
*T*_min_ = 0.933, *T*_max_ = 0.954	*l* = −9→9
6308 measured reflections	3 standard reflections every 97 reflections
2497 independent reflections	intensity decay: 2.3%

### Refinement

**Table:**

Refinement on *F*^2^	Hydrogen site location: inferred from neighbouring sites
Least-squares matrix: full	H-atom parameters constrained
*R*[*F*^2^ > 2σ(*F*^2^)] = 0.060	*w* = 1/[σ^2^(*F*_o_^2^) + (0.0833*P*)^2^ + 0.2257*P*] where *P* = (*F*_o_^2^ + 2*F*_c_^2^)/3
*wR*(*F*^2^) = 0.186	(Δ/σ)_max_ < 0.001
*S* = 1.06	Δρ_max_ = 0.19 e Å^−^^3^
2497 reflections	Δρ_min_ = −0.16 e Å^−^^3^
172 parameters	Extinction correction: *SHELXL97* (Sheldrick, 2008), Fc^*^=kFc[1+0.001xFc^2^λ^3^/sin(2θ)]^-1/4^
0 restraints	Extinction coefficient: 0.036 (6)
0 constraints	Absolute structure: Flack (1983), 1028 Friedel pairs
Primary atom site location: structure-invariant direct methods	Flack parameter: 0.0 (2)
Secondary atom site location: difference Fourier map	

### Fractional atomic coordinates and isotropic or equivalent isotropic displacement parameters (Å^2^)

**Table:**

	*x*	*y*	*z*	*U*_iso_*/*U*_eq_	
N1	0.8943 (5)	0.38124 (15)	1.0214 (4)	0.0789 (10)	
C1	1.0000	0.5000	1.5231 (7)	0.0904 (19)	
H1A	1.0000	0.5000	1.6398	0.108*	
C2	0.9506 (5)	0.4463 (2)	1.4376 (5)	0.0800 (12)	
H2A	0.9166	0.4102	1.4968	0.096*	
C3	0.9508 (4)	0.44531 (18)	1.2633 (5)	0.0660 (10)	
C4	1.0000	0.5000	1.1777 (7)	0.0682 (14)	
H4A	1.0000	0.5000	1.0611	0.082*	
C5	0.9004 (5)	0.38667 (18)	1.1771 (5)	0.0711 (10)	
H5A	0.8711	0.3514	1.2420	0.085*	
C6	0.8370 (5)	0.32020 (18)	0.9537 (5)	0.0747 (11)	
H6A	0.8222	0.2897	1.0464	0.090*	
C7	0.6791 (5)	0.3332 (2)	0.8717 (6)	0.0947 (14)	
H7A	0.6077	0.3499	0.9539	0.142*	
H7B	0.6383	0.2937	0.8262	0.142*	
H7C	0.6918	0.3642	0.7832	0.142*	
C8	0.9507 (5)	0.29178 (16)	0.8309 (5)	0.0646 (9)	
C9	1.0643 (5)	0.32880 (18)	0.7563 (5)	0.0760 (11)	
H9A	1.0740	0.3720	0.7868	0.091*	
C10	1.1656 (6)	0.3034 (2)	0.6361 (6)	0.0924 (14)	
H10A	1.2430	0.3295	0.5899	0.111*	
C11	1.1527 (6)	0.2419 (2)	0.5865 (5)	0.0851 (12)	
H11A	1.2184	0.2263	0.5028	0.102*	
C12	1.0413 (5)	0.20049 (19)	0.6592 (5)	0.0751 (11)	
C13	1.0297 (6)	0.1351 (2)	0.6116 (6)	0.0911 (13)	
H13A	1.0960	0.1188	0.5293	0.109*	
C14	0.9225 (6)	0.0957 (2)	0.6848 (7)	0.0973 (15)	
H14A	0.9158	0.0527	0.6524	0.117*	
C15	0.8233 (5)	0.1194 (2)	0.8073 (7)	0.0909 (14)	
H15A	0.7514	0.0919	0.8578	0.109*	
C16	0.8295 (5)	0.18255 (17)	0.8549 (6)	0.0749 (11)	
H16A	0.7615	0.1975	0.9371	0.090*	
C17	0.9380 (4)	0.22579 (17)	0.7812 (5)	0.0652 (9)	
C18	0.5000	0.5000	0.8916 (9)	0.123 (3)	
H18A	0.4641	0.5349	0.9629	0.148*	
Cl1	0.6505 (3)	0.52641 (11)	0.7706 (3)	0.1852 (10)	

### Atomic displacement parameters (Å^2^)

**Table:**

	*U*^11^	*U*^22^	*U*^33^	*U*^12^	*U*^13^	*U*^23^
N1	0.102 (3)	0.0685 (19)	0.067 (2)	−0.0018 (18)	0.003 (2)	0.0003 (16)
C1	0.099 (5)	0.115 (5)	0.057 (3)	0.000 (4)	0.000	0.000
C2	0.084 (3)	0.090 (3)	0.066 (2)	0.004 (3)	0.008 (2)	0.008 (2)
C3	0.062 (2)	0.076 (2)	0.060 (2)	0.0110 (19)	0.0047 (19)	0.0017 (19)
C4	0.073 (3)	0.075 (3)	0.056 (3)	0.015 (3)	0.000	0.000
C5	0.073 (2)	0.070 (2)	0.070 (3)	0.008 (2)	0.007 (2)	0.008 (2)
C6	0.085 (3)	0.070 (2)	0.069 (2)	−0.001 (2)	0.003 (2)	0.0066 (19)
C7	0.080 (3)	0.099 (3)	0.105 (3)	0.016 (3)	0.008 (3)	0.000 (3)
C8	0.069 (2)	0.064 (2)	0.060 (2)	0.0002 (18)	0.003 (2)	0.0087 (17)
C9	0.085 (3)	0.072 (2)	0.071 (2)	−0.006 (2)	0.005 (2)	0.009 (2)
C10	0.091 (3)	0.100 (3)	0.086 (3)	−0.005 (3)	0.026 (3)	0.022 (3)
C11	0.082 (3)	0.102 (3)	0.072 (3)	0.013 (3)	0.011 (2)	0.003 (2)
C12	0.071 (2)	0.081 (2)	0.073 (2)	0.010 (2)	−0.014 (2)	−0.004 (2)
C13	0.089 (3)	0.090 (3)	0.095 (3)	0.020 (3)	−0.019 (3)	−0.019 (3)
C14	0.097 (3)	0.076 (3)	0.120 (4)	0.012 (3)	−0.030 (4)	−0.010 (3)
C15	0.078 (3)	0.075 (3)	0.120 (4)	−0.005 (2)	−0.024 (3)	0.007 (3)
C16	0.066 (2)	0.072 (2)	0.087 (3)	−0.004 (2)	−0.004 (2)	0.002 (2)
C17	0.059 (2)	0.069 (2)	0.068 (2)	0.0022 (18)	−0.007 (2)	0.0072 (18)
C18	0.192 (10)	0.094 (5)	0.084 (4)	−0.003 (5)	0.000	0.000
Cl1	0.1454 (16)	0.208 (2)	0.202 (2)	−0.0442 (14)	0.0100 (17)	0.0547 (16)

### Geometric parameters (Å, °)

**Table:**

N1—C5	1.248 (5)	C9—C10	1.395 (6)
N1—C6	1.459 (5)	C9—H9A	0.9300
C1—C2	1.371 (5)	C10—C11	1.338 (6)
C1—C2^i^	1.371 (5)	C10—H10A	0.9300
C1—H1A	0.9300	C11—C12	1.407 (6)
C2—C3	1.390 (5)	C11—H11A	0.9300
C2—H2A	0.9300	C12—C13	1.410 (6)
C3—C4	1.387 (4)	C12—C17	1.414 (5)
C3—C5	1.460 (5)	C13—C14	1.359 (7)
C4—C3^i^	1.387 (4)	C13—H13A	0.9300
C4—H4A	0.9300	C14—C15	1.384 (7)
C5—H5A	0.9300	C14—H14A	0.9300
C6—C8	1.500 (5)	C15—C16	1.363 (5)
C6—C7	1.524 (6)	C15—H15A	0.9300
C6—H6A	0.9800	C16—C17	1.416 (5)
C7—H7A	0.9600	C16—H16A	0.9300
C7—H7B	0.9600	C18—Cl1^ii^	1.699 (5)
C7—H7C	0.9600	C18—Cl1	1.699 (5)
C8—C9	1.373 (5)	C18—H18A	0.9698
C8—C17	1.427 (5)		
			
C5—N1—C6	117.4 (3)	C8—C9—C10	121.8 (4)
C2—C1—C2^i^	120.4 (6)	C8—C9—H9A	119.1
C2—C1—H1A	119.8	C10—C9—H9A	119.1
C2^i^—C1—H1A	119.8	C11—C10—C9	120.7 (4)
C1—C2—C3	120.6 (4)	C11—C10—H10A	119.7
C1—C2—H2A	119.7	C9—C10—H10A	119.7
C3—C2—H2A	119.7	C10—C11—C12	121.0 (4)
C4—C3—C2	118.7 (4)	C10—C11—H11A	119.5
C4—C3—C5	122.5 (4)	C12—C11—H11A	119.5
C2—C3—C5	118.8 (4)	C11—C12—C13	121.5 (4)
C3—C4—C3^i^	121.1 (5)	C11—C12—C17	118.7 (4)
C3—C4—H4A	119.4	C13—C12—C17	119.8 (4)
C3^i^—C4—H4A	119.4	C14—C13—C12	120.5 (5)
N1—C5—C3	123.7 (4)	C14—C13—H13A	119.7
N1—C5—H5A	118.1	C12—C13—H13A	119.7
C3—C5—H5A	118.1	C13—C14—C15	120.3 (4)
N1—C6—C8	111.3 (3)	C13—C14—H14A	119.9
N1—C6—C7	107.6 (3)	C15—C14—H14A	119.9
C8—C6—C7	111.3 (3)	C16—C15—C14	120.8 (5)
N1—C6—H6A	108.8	C16—C15—H15A	119.6
C8—C6—H6A	108.8	C14—C15—H15A	119.6
C7—C6—H6A	108.8	C15—C16—C17	121.1 (4)
C6—C7—H7A	109.5	C15—C16—H16A	119.4
C6—C7—H7B	109.5	C17—C16—H16A	119.4
H7A—C7—H7B	109.5	C12—C17—C16	117.4 (4)
C6—C7—H7C	109.5	C12—C17—C8	119.9 (3)
H7A—C7—H7C	109.5	C16—C17—C8	122.7 (4)
H7B—C7—H7C	109.5	Cl1^ii^—C18—Cl1	110.8 (4)
C9—C8—C17	117.9 (4)	Cl1^ii^—C18—H18A	109.4
C9—C8—C6	121.5 (3)	Cl1—C18—H18A	109.4
C17—C8—C6	120.5 (3)		
			
C2^i^—C1—C2—C3	0.4 (3)	C10—C11—C12—C13	−178.1 (4)
C1—C2—C3—C4	−0.9 (6)	C10—C11—C12—C17	2.7 (6)
C1—C2—C3—C5	178.7 (3)	C11—C12—C13—C14	179.2 (4)
C2—C3—C4—C3^i^	0.4 (3)	C17—C12—C13—C14	−1.6 (6)
C5—C3—C4—C3^i^	−179.1 (4)	C12—C13—C14—C15	0.0 (7)
C6—N1—C5—C3	−178.2 (4)	C13—C14—C15—C16	1.0 (7)
C4—C3—C5—N1	−1.5 (6)	C14—C15—C16—C17	−0.3 (6)
C2—C3—C5—N1	179.0 (4)	C11—C12—C17—C16	−178.6 (4)
C5—N1—C6—C8	−126.4 (4)	C13—C12—C17—C16	2.2 (5)
C5—N1—C6—C7	111.4 (4)	C11—C12—C17—C8	−1.5 (5)
N1—C6—C8—C9	−19.0 (5)	C13—C12—C17—C8	179.3 (4)
C7—C6—C8—C9	101.1 (4)	C15—C16—C17—C12	−1.3 (6)
N1—C6—C8—C17	164.5 (3)	C15—C16—C17—C8	−178.3 (4)
C7—C6—C8—C17	−75.4 (4)	C9—C8—C17—C12	0.3 (5)
C17—C8—C9—C10	−0.3 (6)	C6—C8—C17—C12	177.0 (3)
C6—C8—C9—C10	−176.9 (4)	C9—C8—C17—C16	177.2 (3)
C8—C9—C10—C11	1.5 (7)	C6—C8—C17—C16	−6.1 (5)
C9—C10—C11—C12	−2.7 (7)		

Symmetry codes: (i) −*x*+2, −*y*+1, *z*; (ii) −*x*+1, −*y*+1, *z*.
